# The Effects of Polyphenol Supplementation on BDNF, Cytokines and Cognition in Trained Male Cyclists following Acute Ozone Exposure during High-Intensity Cycling

**DOI:** 10.3390/nu16020233

**Published:** 2024-01-11

**Authors:** Lillian Morton, Carl Paton, Andrea Braakhuis

**Affiliations:** 1 Department of Nutrition, Faculty of Medical & Health Science, The University of Auckland, Auckland 1023, New Zealand; a.braakhuis@auckland.ac.nz; 2 School of Health and Sport Science, The Eastern Institute of Technology, Napier 4142, New Zealand; cpaton@eit.ac.nz

**Keywords:** BDNF, cognition, polyphenols, exercise, ozone

## Abstract

The neurotoxic effects of ozone exposure are related to neuroinflammation and increases in reactive oxygen species (ROS). This study aimed to assess inflammation, Brain-Derived Neurotrophic Factor (BDNF), and cognition in healthy male cyclists following polyphenol supplementation and exercise in an ozone-polluted environment. Ten male cyclists initially completed a maximal incremental test and maximal effort 4 km time trial in ambient air. Cyclists then completed two trials in an ozone-polluted environment (0.25 ppm) following 7 days of supplementation with either polyphenol (POLY) or placebo (PL). Experimental trials consisted of a three-stage submaximal test followed by a 4 km time trial. Blood samples were drawn pre- and post-exercise, and analyzed for BDNF, interleukin 6 (IL-6), interleukin 10 (IL-10) and tumor necrosis factor (TNF-α). The Stroop test and serial subtraction task were performed before ozone exposure and again after the 4 km TT. Serum BDNF increased post-exercise (*p* < 0.0001), and positive differences were observed post-exercise in the ozone POLY group relative to PL (*p* = 0.013). Plasma IL-6 increased post-exercise (*p* = 0.0015), and TNF-α increased post-ozone exposure (*p* = 0.0018). There were no differences in Stroop or serial subtraction tasks pre- or post-exercise. Exercise increases BDNF in ozone.

## 1. Introduction

Tropospheric ozone is an environmental component of urban air pollution. It is generated photochemically with hydrocarbons, nitrogen oxides, and volatile organic compounds (VOCs) [1,2]. Ozone is a highly reactive oxidizing molecule linked to dysfunction of the respiratory and cardiovascular systems [3,4,5,6,7]. The adverse effects of ozone exposure on the central nervous system (CNS) have been described, and ozone exposure is associated with neurological disorders such as Alzheimer’s and Parkinson’s disease [7,8], cognitive impairment [9,10], dementia [11], and neuroinflammation [12].

The neurotoxic effects of ozone exposure on the CNS have been established in both human and animal studies. Animal models suggest that ozone significantly interferes with neural physiology by increasing lipid peroxidation, reducing dopaminergic neurons, increasing interleukin-6 (IL-6) and tumor necrosis factor α (TNF-α), and accumulating amyloid-b and a-synuclein, which are pathologic proteins in Alzheimer’s and Parkinson’s disease respectively [7,12,13,14,15]. Compared to other structures, the hippocampus is differentially sensitive to the oxidative effects of ozone [6] and is the first region to be affected by acute ozone exposure [7], due to the connection of the olfactory bulbs and nerves to the hippocampus, the varying antioxidant capacity of brain regions, or a combination of these [7].

Brain-derived neurotrophic factor (BDNF) is found throughout the brain and abundantly in the hippocampus [16]. BDNF plays a role in neuronal growth, differentiation, neuronal survival, synaptic plasticity, and memory and learning processes [16,17]. Human and animal studies have demonstrated that acute exercise bouts stimulate neuronal function, brain vascularization, and neurogenesis, improve mood, and enhance learning [18,19] via the elevation of BDNF [18,19,20,21,22,23]. Conversely, exercise in polluted air has been shown to inhibit the acute exercise-induced increase in BDNF secretion typically seen following exercise bouts, suggesting that exposure to air pollution blunts the improvement in cognitive health and CNS plasticity [18,19,20].

Epidemiological and randomized control trials have shown that an increased intake of polyphenols can be linked to neuroprotective effects and improved cognitive function [24,25,26,27,28,29,30,31,32,33], as well as reduced inflammation in the respiratory system [34,35,36,37,38,39,40,41,42]. Phenolic acids and phenolic compounds have also been reported to have a beneficial effect on BDNF levels [25].

Controlled trials examining the acute effects of exercise on BDNF with ozone exposure in human subjects are lacking, particularly in the context of using a nutritional intervention to augment the effects. This study therefore investigated whether supplementation with a functional blend of phytonutrients containing blackcurrant, L-theanine, and pine bark extract has a neuroprotective effect and affects cognitive processes following high-intensity interval exercise performed during ozone exposure.

## 2. Materials and Methods

### 2.1. Participants

Ten healthy male cyclists gave written informed consent prior to participation in the study (mean ± SD: age, 43.8 ± 12.38 years; height, 177.8 ± 7.1 cm; weight, 76.03 ± 7.88 kg; $\dot{V}$O_2max_ 4.12 ± 0.72 L·min^−1^). Participants were required to avoid strenuous training and activity in the 24 h preceding testing, but light activity or training was allowed. Dietary intakes over a 24 h period were recorded by participants before the first trial. Physical activity, light training, and nutrition were replicated prior to each testing session. Participants refrained from eating and drank only plain water in the two hours before the commencement of each testing session. All trials were attended at the same time of day (±90 min) to control for any potential diurnal variations. The study was conducted according to the guidelines of the Declaration of Helsinki, approved by the Northern Health and Disability Ethics Committee (ref 21/NTB/68, 19 April 2021), and registered with the Australia New Zealand Clinical Trial Registry (ACTRN12622001198718).

### 2.2. Experimental Design

**Figure 1:**
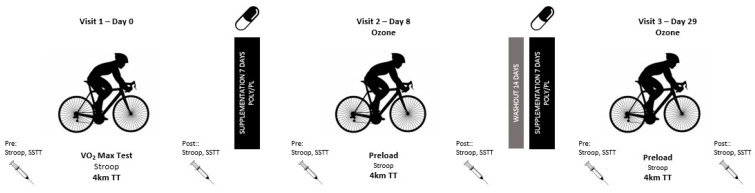
Study design and data collection points.

The study was a randomized, double-blind, placebo-controlled, crossover design. Participants visited the laboratory on three separate occasions. An initial visit to determine maximal oxygen consumption ($\dot{V}$O_2max_) and peak power output, and familiarize the participants with the cognitive testing procedures and equipment, and was completed in ambient air. Following this, participants reported to the laboratory for two trials performed in an ozone-polluted environment after 7 d of supplementation with either polyphenol (POLY + ozone) or placebo (PL + ozone). A washout period of 14 d occurred between each supplementation period. The same researcher supervised all trials and was blinded to the experimental conditions. A summary of the study design is shown in Figure 1.

### 2.3. Ozone Exposure

An ozone concentration of 0.25 ppm was generated by a silent discharge method using an ozone generator (N50-C Ozone Generator). An ozone concentration within ±10% of 0.25 ppm was automatically maintained by a relay sensor (airQual, SM-70, Novozone, Auckland, New Zealand).

### 2.4. Experimental Trial

Full details of the exercise testing regimes and exercise endpoints have been reported elsewhere [43]. In brief, subjects were randomly assigned in a double-blind crossover design to receive either PL or POLY for 7 days prior to exercise and cognitive testing. The POLY supplement was a commercially available combination of freeze-dried NZ blackcurrant (94%; Delphinidin-3-0-Glucoside, 176 mg/5 g, Delphinidin-3-0-Rutinoside, 752 mg/5 g, Cyanidin-3-0-Glucoside, 84 mg/5 g, Cyanidin-3-0-Rutinoside, 722 mg/5 g, Total Anthocyanins, 1733.5 mg/5 g), L-theanine (3%), and Pinus radiata extract (3%) (Ᾱrepa™), and was prescribed as a relative dose of 4.3 mg anthocyanins/kg^−1^. A berry-flavored powder that contained no vitamin C, active ingredients, or phenolics was used as the PL [43]. The POLY and PL were equivalent in energy and carbohydrate content (per 5 g—POLY: 15 cal, carbohydrate 3 g, PL: 16 cal, carbohydrate 3.6 g). Both the POLY and PL were encapsulated into purple, berry-flavored gelatin capsules and delivered in opaque unmarked bottles to maintain the integrity of the anthocyanins. An independent person gave either POLY or PL to the researcher on the day of testing for distribution to participants. To determine adherence to the supplementation protocol, participants were oversupplied with capsules and a manual countback of returned capsules was completed.

Following 7 days of supplementation, participants completed the experimental protocol [43]. Participants were weighed when they arrived at the laboratory, and blood samples were drawn. Participants then completed the Stroop test and serial 3 and serial 7 subtraction tasks and fitted with measurement equipment. All cycling trials were performed on a calibrated Velotron Dynafit Pro cycle ergometer (RacerMate Inc., Seattle, WA, USA) using the company’s associated software package. Participants performed a warm-up for 5 min before completing a cycling protocol consisting of 10 min at 50% peak power output (PPO), 10 min at 60% PPO, and 5 min at 70% PPO. PPO was determined at the participant’s initial visit. The cycling load was automatically controlled by the ergometer. A 10 min rest followed the cycling protocol, and participants completed the Stroop test in a seated position within the chamber during the rest period. Participants were then required to complete a maximal effort 4-km TT in the shortest time possible. After completion of the 4-km TT, participants dismounted the ergometer. In a seated position within the chamber, a blood sample was drawn, and participants then completed the Stroop test and serial 3’s and 7’s subtraction task. Participants were permitted to drink water ad libitum in the rest period and after the 4-km TT.

### 2.5. Blood Sampling

Venous blood samples were drawn from an antecubital vein 30 min before exercise and 5 min after the completion of the 4-km TT, as BDNF levels have been shown to return to baseline levels within 20 min after exercise [44]. At each sampling point, 5 mL of blood was drawn into a coagulant-free serum separator tube (SST Tube), and 5 mL was drawn into a vacutainer containing ethylenediaminetetraacetic acid (EDTA) (Becton, Dickinson and Company, Franklin Lakes, NJ, USA). Blood samples were left to clot at room temperature for 20 min and then centrifuged at 1000× *g* for 10 min to separate serum and plasma. The resulting supernatant was then aliquoted into Eppendorf tubes and stored at −20 °C until analysis.

### 2.6. Sample Analysis

#### 2.6.1. BDNF

BDNF levels were determined from serum using a quantitative sandwich enzyme-linked immunosorbent assay (ELISA) kit (Quantikine Human Free BDNF ELISA, R&D Systems, Biotechne, Minneapolis, MN, USA) according to the manufacturer’s protocol. All samples were diluted 20x prior to analysis (10 µL of sample to 190 µL of calibrator Diluent RD6P). The coefficients of variation for BDNF were 7.37% at low levels (233 ± 17.18 pg/mL), 2.04% at medium levels (896 ± 18.24 pg/mL) and 5.23% at high levels (1946 ± 101.82 pg/mL) of control material.

#### 2.6.2. IL-6, IL-10 and tnf-α

Plasma cytokine IL-6, IL-10 and TNF-α levels were assessed using the multiplex bead-based immunoassay Luminex system using the Milliplex^®^ MAP Human Cytokine/Chemokine/Growth Factor panel (EMD Millipore, Billerica, MA, USA) according to the manufacturers’ protocol. Analytes included IL-6, IL-10, and TNF-α. Twenty-five microliters of neat plasma sample was used in the wells, and the plate was sealed and incubated with agitation on a plate shaker overnight (16–18 h) at 2–8 °C. Data were analyzed using Luminex xPonent 3.1 software. The average CVs of quality control were 6.30 ± 4.26% for IL-6, 13.57 ± 15.68% for IL-10 and 8.03 ± 2.71% for TNF-α.

### 2.7. Cognitive Tests

Participants completed a Stroop test and serial 3’s (SST3) and serial 7’s subtraction task (SST7) pre- and post-exercise. The pre-test was completed before entry into the ozone chamber in a quiet room with no distractions. Post-testing was completed 15 min post-exercise and conducted in the ozone chamber.

#### 2.7.1. Stroop Test

The Stroop test was administered on an iPad using a validated app-based version (EncephelApp), which has been shown to have high test-retest reliability with an intraclass coefficient of 0.83 [45]. The task has an easier ‘OFF’ state where subjects selected the appropriate color of hashtag symbols (###) presented in one of four colors ‘RED’, ‘GREEN’, ‘BLUE’ ‘YELLOW’ as fast as possible by selecting the matching color of the stimulus at the bottom of the screen. Subjects performed one training run and one trial of 10 stimuli presentations. If an incorrect color was selected, the run was stopped and restarted. In the ’ON’ state of the test, incongruent stimuli are presented in 9 of the 10 stimuli. Words describing one of four colors were randomly presented where the word and font were different colors (i.e., the word ‘RED’ is displayed in blue). Subjects had to select the correct color of the word, not the name (i.e., selecting blue in the preceding example), by selecting the correct color at the bottom of the screen as fast as possible. Subjects completed one practice run and three trials of 10 stimuli presentations. Both the stimuli and the response buttons were randomized and not fixed in their respective positions each time. The average reaction time and error rate for each trial were calculated.

#### 2.7.2. Serial Subtraction Task (3’s and 7’s)

Serial 3’s and 7’s subtraction tasks are used to assess attention or working memory function [46]. In brief, participants were required to sequentially subtract 7 from 100 and continue subtracting 7 from their answer within a given time. The serial 3’s are similar in nature but start at 50. The protocols used in the present study are based on those of Bistow et al. [46], where subjects had 20 s to complete as many answers as possible. To minimize any potential learning effect, the serial 3 start numbers in the pre-test were 60 and 51 post-exercise in trial 1, and 80 and 76 pre- and post-exercise respectively in trial 2. The start numbers for the serial 7 numbers were 71 pre-trial and 82 post-exercise in trial 1 and 130 and 110 in trial 2.

The examiner read the following instructions to the participant:

“I want you to perform a mental, cognitive task that requires subtracting. I am going to say a number and I would like you to subtract 3/7 from that number and continue to subtract 3/7 from your answer until I tell you to stop. Work as quickly as you can and try not to make any mis-takes. Any questions? Ready? Here is the number XX”.

The responses of the participant were recorded by the researcher. Correct calculations of 3 or 7 were scored as a correct response. If another number other than 3 or 7 was given, it was recorded as an error. The total number of correct responses and number of errors were used for data analysis.

### 2.8. Data Analysis Procedures and Statistical Analysis

Statistical analyses were conducted using GraphPad Prism version 9.3.0 for Windows (GraphPad Software, San Diego, CA, USA). The normality of data residuals was confirmed using the Shapiro–Wilk test prior to all analyses. Where normality was violated, data were log-transformed. Differences between treatments (treatment × time) were determined using a two-way repeated measures analysis of variance (ANOVA), and Šídák’s multiple comparisons test post hoc analysis was used where significance was detected to compare differences between treatments. Given the potential for learning effects, the data from Stroop and serial subtraction tasks in the familiarization trials were excluded from analysis.

Due to technical issues with blood samples, six samples were missing from a total of 40 samples under ozone exposure. Plasma cytokine analyses were therefore analyzed using a restricted maximum likelihood (REML) mixed-effects model to account for the missing values. To determine the goodness of fit of the REML model, visual assessment of the residual QQ plots was undertaken to check for deviations. The fixed effects (type III) in the model were time, treatment, and time × treatment. Statistical significance was defined as *p* ≤ 0.05. Šídák’s multiple comparisons test post hoc analysis was used to compare differences between treatments. The magnitudes of the standardized effects for the test measures were determined using the Cohen effect size (d). Thresholds of 0.2, 0.5, and 0.8 for small, moderate, and large effects, respectively, were used in accordance with the recommendations of Cohen [47]. ES values < 0.2 were deemed trivial differences. Simple group statistics are shown as the means ± SDs, unless otherwise stated. Percentage (%) differences between treatments are reported as the mean ± 95% confidence intervals (CI).

## 3. Results

### 3.1. BDNF

**Figure 2:**
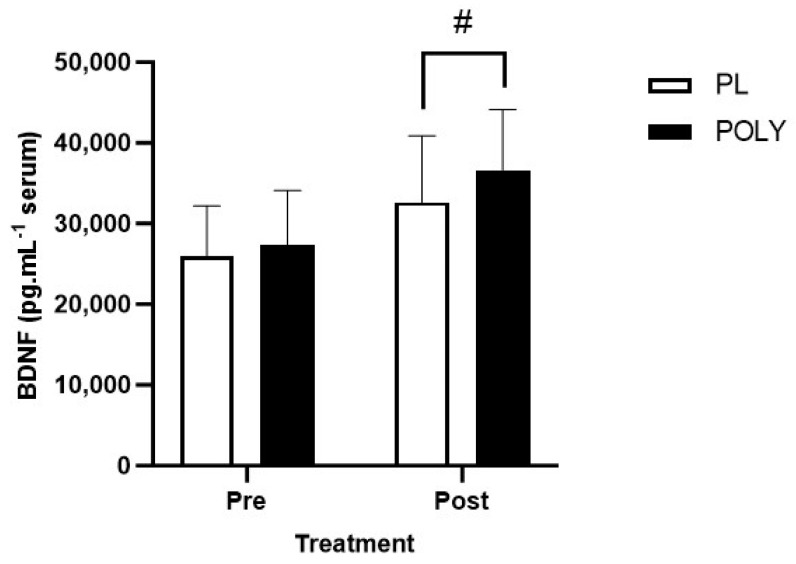
Serum BDNF levels pre- and post-exercise in ozone following 7 days of supplementation of either PL or POLY. Data presented as mean ± SD. ^#^ *p* = 0.019 ^#^ denotes a treatment effect.

BDNF levels increased in both the PL and POLY treatment arms following exercise in an ozone-polluted environment (main time effect *p* < 0.0001). A main treatment effect was observed in BDNF levels between PL and POLY (*p* = 0.013). Šídák’s post hoc analysis revealed a significant difference and a small effect (*p* = 0.019, −3523 95% CI −6479 to −566.9, d = 0.25) between PL (34,732.50 ± 7833.30 8263.92 pg/mL) and POLY (36,638.70 ± 7493.77 pg/mL) post-exercise (Figure 2).

### 3.2. IL-6, IL-10 and TNF-α

**Figure 3:**
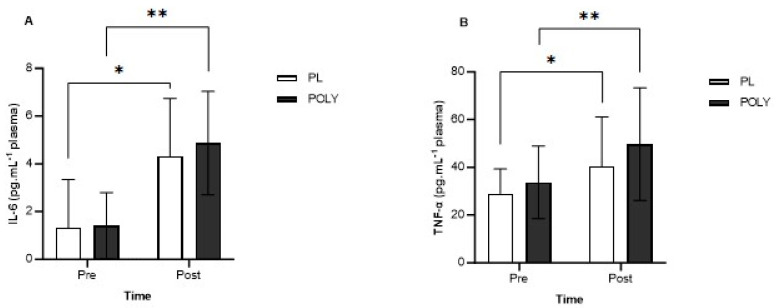
(**A**) Plasma IL-6 (pg/mL) pre- and post-exercise. * *p* = 0.001, ** *p* = 0.019. (**B**) Plasma TNF-α plasma levels (pg/mL) pre- and post-exercise in ozone. * *p* = 0.005, ** *p* = 0.016. Data presented as mean ± SD. * denotes an effect for time.

There was a significant main effect for time in plasma IL-6 levels (*p* = 0.0015, F_(1, 9)_ = 21.94). IL-6 increased post-exercise (PL: 1.33 ± 2.01 to 4.32 ± 2.42 pg/mL, *p* = 0.001, POLY: 1.41 ± 1.39 to 4.87 ± 2.17 pg/mL, *p* = 0.019) regardless of supplementation (Figure 3A). A significant time effect (*p* = 0.0018, F_(1, 7)_ = 23.65) was observed in plasma TNF-α (Figure 3B). Post hoc analysis revealed significantly higher TNF-α levels post exercise in ozone. TNF-α levels increased from 29.03 ± 10.24 to 40.34 ± 20.78 pg/mL in the PL arm (*p* = 0.005), and 33.74 ± 15.16 to 49.72 ± 23.58 pg/mL following POLY supplementation (*p* = 0.016). No statistical differences were observed between PL and POLY TNF-α levels, however small effects were seen for POLY treatment both pre (d = 0.36) and post (d = 0.42) exercise. No significant changes in plasma IL-10 were observed between treatments (*p* = 0.89), but a significant main effect for time was observed (*p* = 0.016). Plasma IL-10 increased post exercise in both PL (pre: 5.56 ± 3.78, post: 7.33 ± 3.66, *p* = 0.06) and POLY (pre: 4.99 ± 2.90, post: 9.05 ± 5.61 pg/mL, *p* = 0.016).

### 3.3. Stroop Test

There were no differences in Stroop performance before or after exercise in an ozone-polluted environment (main time effect *p* = 0.22) or between PL and POLY treatments either pre-exercise (*p* = 0.83, d = 0.19), post-preload (*p* => 0.99, d = 0.03) or post the 4 km TT (*p* > 0.99, d = 0.03). The number of errors made was not significantly different in the PL and POLY conditions pre-exercise (*p* = 0.82, d = 0.44), post the preload (*p* = 0.97, d = 0.20), or following the 4 km TT (*p* = 0.22, d = 0.22). Means ± SDs for the Stroop task are shown in Table 1.

### 3.4. Serial Subtraction Task

A significant main effect for treatment (*p* = 0.05) was observed in the number of responses in the serial subtraction 3’s task. However, post hoc analysis showed no treatment differences between PL or POLY either pre- (−0.40, 95% CI −1.93 to 1.13, *p* = 0.75, d = 0.13) or post (−0.80, 95% CI −2.34 to 0.73, *p* = 0.35) exercise in ozone. There was a significant main effect for time in the serial 7’s task (*p* = 0.03), but no significant differences were seen with post hoc analysis (Pre: −1.70, 95% CI −4.09 to 0.69, *p* = 0.17, d = 0.45, Post: −1.40, 95% CI −3.79 to 0.99, *p* = 0.28, d = 0.34). There was a significant reduction in errors in the serial 3’s task with POLY treatment post-exercise (*p* = 0.004), but no differences in error rate between PL and POLY treatments either pre- (*p* > 0.99, d = 0.00) or post- (*p* = 0.12, d = 0.67) exercising in ozone in the serial 3 task or the serial 7 s (pre: *p* = 0.61, d = 0.27; post: *p* = 0.94, d = 0.08). Means ± SDs for serial subtraction task measures are shown in Table 2.

## 4. Discussion

The primary finding of the present study was that serum BDNF levels increased following an acute bout of high-intensity exercise in an ozone-polluted environment. In addition, supplementation with an anthocyanin-rich supplement providing 4.3 mg/kg anthocyanins daily for 7 days resulted in significantly higher BDNF levels post-exercise compared to PL. Plasma IL-6 levels in the present study increased in participants post exercise, including the familiarization trial performed under ambient air, while the levels of TNF-α increased following cycling in ozone. There were no significant improvements in cognitive tasks typically seen following exercise.

Performance in the Stroop test and serial subtraction tasks in the present study did not differ between pre- and post-cycling in ozone in either treatment arm, suggesting a lack of improvement in cognition after exercise in ozone. However, small, nonsignificant effects were observed in the serial subtraction tasks, with a higher number of responses post-exercise and a higher response rate pre-exercise in the POLY group. The acute effects of ozone exposure on cognition are not well established, but adverse effects on cognitive tasks related to short-term memory and attention were consistently observed in a cohort of healthy adults aged 20–50 years after ozone exposure [48]. While cognitive test performance in the present study did not worsen post-exercise, the lack of improvement indicates a potential negative effect of ozone on cognitive domains assessing attention and executive function.

Previous work by Bos et al. [18] found that BDNF increased ~14% after exercise in clean air, but no elevations in BDNF were seen after cycling along a busy road. BDNF levels in the present study increased 29% following exercise in ozone in the PL arm and 35% with polyphenol supplementation and exercise. Evidence suggests a dose–response relationship between acute exercise and BDNF levels [49], with acute high-intensity exercise bouts and graded exercise tests eliciting higher BDNF levels post exercise in healthy subjects. The present study utilized a preload protocol relative to each individual’s cycling ability (as measured by peak power output). Cyclists therefore increased their exercise intensity progressively over a 25-min period and then cycled maximally for 4 km. This exercise protocol had higher maximal heart rates and a longer duration (approximately 65 min exposure vs. 20 min) than those in the Bos et al. [18] study. Our findings are in alignment with those of Silveira et al. [50]. While the cyclists in their study were not supplemented, they did show elevations in BDNF after completing a 50 km TT in traffic-related air pollution.

While IL-6 is used to reflect inflammation, it is also now recognized as a myokine, and plasma levels of IL-6 increase during muscular work [51]. In the present study, there was a 3-fold increase in plasma IL-6 levels following exercise regardless of air quality or supplementation, indicating that the increases in IL-6 in this case may be attributed to the exercise protocol independent of ozone exposure. The release of IL-6 stimulates the secretion of the anti-inflammatory IL-10 [52]. In the present study, a significant difference was observed in IL-10 levels post-exercise in ozone with POLY supplementation. Typically, plasma levels of TNF-α do not increase as a result of exercise [53], but elevations in TNF-α have been observed in both bronchoalveolar lavage fluid (BALF) and the cerebral cortex following 6 h of ozone exposure in mice [54]. In the present study, the levels of TNF-α increased following cycling in ozone but not in ambient air. The increase in TNF-α can then be attributed to ozone exposure during exercise, and elevated levels may reflect systemic inflammation and activation of the innate immune system [55]. Ozone exposure can increase the levels of TNF-α, disrupt blood brain barrier permeability, and result in neuroinflammation [56]. In the present study, supplementation with a polyphenol powder providing anthocyanins for 7-days preceding the exercise trials did not attenuate the increase in TNF-α.

Ozone exposure is known to cause oxidative stress, and the disruption to the oxidation–reduction balance leads to progressive damage and causes alterations in the hippocampus at molecular, cellular, and cognitive levels [2,57]. Serum BDNF is known for its role in synaptic plasticity [58], as it regulates the survival and differentiation of neurons and influences the formation of dendrites and dendritic spindles [59]. A systematic review by Gravesteijn, Mensink, and Plat [25] found significant improvement in BDNF in four of 11 studies. However, exercise was not a component in any of these studies. To our knowledge, no other research has explored the effect of a combination of exercise and polyphenol consumption on BDNF levels. The results from the present study show that supplementation of an anthocyanin-rich polyphenol powder combined with exercise elevates BDNF levels after cycling in ozone.

A limitation of the study is the small sample size. Thirteen subjects were initially recruited. Two subjects withdrew due to pronounced symptomatic responses to ozone exposure and another due to work commitments. Furthermore, five of 40 samples were missing for plasma cytokine analysis. We recommend that future studies be conducted to confirm the findings from the present study, and that a larger sample size be recruited. While our subjects performed exercise under conditions of ozone pollution, the effects of polyphenol supplementation combined with exercise in ambient air remains unknown.

## 5. Conclusions

The results of the present study indicate that exercise combined with polyphenol supplementation increases BDNF levels in healthy cyclists exercising in poor air quality.

## Figures and Tables

**Table 1:** Stroop test performance before and after exercise in ozone following either PL or POLY supplementation.

	PL			POLY		
	Pre	Post 1	Post 2	Pre	Post 1	Post 2
Stroop speed (s)	13.63 ± 3.07	13.18 ± 3.27	13.48 ± 3.26	14.26 ± 3.65	13.10 ± 3.02	13.58 ± 3.17
Stroop time (s)	27.26 ± 6.14	26.37 ± 6.53	26.96 ± 6.52	28.55 ± 7.33	26.77 ± 7.13	27.66 ± 7.02
Stroop errors	0.20 ± 0.63	0.10 ± 0.32	0.30 ± 0.48	0 ± 0	0.20 ± 6.32	0.20 ± 0.42

Data reported as the mean ± SD from 10 subjects.

**Table 2:** Serial subtraction 3’s and 7’s task performance before and after exercise in ozone following 7 days of supplementation with PL or POLY.

	PL		POLY	
	Pre	Post	Pre	Post
Serial 3 s	13.80 ± 3.43	14.20 ± 2.20	14.20 ± 2.62	15.00 ± 2.31
Serial 7 s	6.40 ± 3.78	7.70 ± 4.73	8.10 ± 3.84	9.10 ± 3.70
Serial 3 errors	0.40 ± 0.84	0.20 ± 0.42	0.40 ± 0.84	0.0 ± 0.0 *
Serial 7 errors	0.80 ± 0.79	0.90 ± 1.01	0.50 ± 0.71	1.00 ± 1.25

Data reported as the mean ± SD from 10 subjects. Serial = Serial Subtraction Tasks. * *p* = 0.004 pre to post exercise.

## Data Availability

Data are contained within the article.
